# Alpha-1 Antitrypsin Deficiency Beyond COPD and Emphysema: A Narrative Review

**DOI:** 10.3390/medsci14010106

**Published:** 2026-02-22

**Authors:** Lucia Pastoressa, Vanessa Pivetti, Marialuisa Valente, Bianca Beghè, Enrico Clini, Roberto Tonelli, Stefania Cerri

**Affiliations:** 1 Department of Medical and Surgical Sciences, University of Modena and Reggio Emilia, 41124 Modena, Italy; luciapastoressa2@gmail.com (L.P.); 211797@studenti.unimore.it (V.P.); ma.valenteee@gmail.com (M.V.); bianca.beghe@unimore.it (B.B.); enrico.clini@unimore.it (E.C.); roberto.tonelli@unimore.it (R.T.); 2 Respiratory Diseases Unit, University Hospital of Modena—Policlinico, 41124 Modena, Italy; 3 Center for Rare Lung Diseases, University Hospital of Modena—Policlinico, 41124 Modena, Italy

**Keywords:** alpha-1 antitrypsin, chronic obstructive pulmonary disease, emphysema, asthma, bronchiectasis, neutrophil elastase, protease–antiprotease imbalance

## Abstract

**Background/Objectives**: Alpha-1 antitrypsin deficiency (AATD) is a genetic disorder classically associated with emphysema and COPD. However, emerging evidence indicates that its clinical spectrum extends to airway-predominant diseases such as bronchiectasis and asthma, where protease–antiprotease imbalance and neutrophilic inflammation may drive tissue injury. This narrative review aims to synthesize current evidence on the relationship between AATD and airway diseases beyond emphysema, focusing on epidemiological patterns, underlying mechanisms, diagnostic strategies, and therapeutic implications. **Methods**: A narrative synthesis of the literature was performed, integrating data from registries, with observational and translational studies addressing the prevalence, pathobiology, and therapeutic implications of AATD in bronchiectasis, asthma, and severe asthma. Epidemiologic and mechanistic insights were analyzed to identify overlapping pathways and evidence gaps. **Results:** Evidence supports a non-negligible prevalence of bronchiectasis and asthma among AATD individuals, particularly in severe or heterozygous genotypes. Neutrophil elastase overactivity, impaired mucociliary clearance, and chronic neutrophilic inflammation emerge as shared mechanisms promoting bronchial remodeling and airflow limitation. In asthma, AATD appears linked to T2-low, steroid-resistant phenotypes and persistent obstruction, whereas in severe asthma cohorts, up to 20% may carry non-PiMM SERPINA 1 variants. No randomized trials have evaluated augmentation therapy and standardized screening algorithms are lacking. **Conclusions**: AATD represents a systemic disorder with clinically relevant airway manifestations beyond COPD and emphysema. Targeted testing should be considered in patients with idiopathic bronchiectasis or severe asthma. Future genotype-stratified, prospective studies are required to clarify causality, define biomarkers of disease activity, and evaluate the potential role of anti-protease-based therapeutic strategies.

## 1. Introduction and Methodology

### 1.1. Introduction

Alpha-1 antitrypsin (AAT)—a serpin produced primarily by hepatocytes and, to a lesser extent, by myeloid and epithelial cells—inhibits neutrophil elastase (NE) and other serine proteases, thereby preserving the pulmonary extracellular matrix and tissue homeostasis [1,2]. Beyond its antiprotease role, AAT is an acute-phase protein whose plasma levels rise rapidly during inflammation via IL-6–mediated hepatic up-regulation [1,3]. It also exerts immunomodulatory activity, dampening innate and adaptive inflammatory responses [1]. Pathophysiologically, AAT is a central regulator of the protease–antiprotease balance: insufficient quantity or function shifts the axis toward proteolysis, promoting extracellular matrix injury, airway remodeling, and emphysema [3,4].

Alpha-1 antitrypsin deficiency (AATD) is a genetic disorder caused by mutations in the *SERPINA1* gene, leading to reduced circulating levels and/or impaired function of the protein. More than 120 genetic variants of *SERPINA1* have been described, which are traditionally classified into normal (M alleles), deficient (S and Z alleles), null (absence of protein production), and dysfunctional variants [5]. The most relevant pathogenic variants are the Z (Glu342Lys) and S (Glu264Val) alleles, which may be present in homozygous or heterozygous forms [6]. For the purpose of this review AATD-related variants have been categorized into two clinically distinct groups: clinically significant AATD (e.g., PiZZ, PiNull and selected PiSZ genotypes and/or serumAAT levels< 11 µM ore <57 mg/dL) and heterozygous carrier status (PiMZ and PiMS genotypes associated with intermediate AAT levels and variable clinical impact). Throughout our review prevalence data are interpreted in consideration of cohort characteristics and testing modality (serum AAT measurements, phenotyping on genotyping). The disease is characterized by a dual pathogenic mechanism: a loss of function, due to insufficient inhibition of NE with subsequent proteolytic damage in the lung, and a toxic gain of function, related to intracellular accumulation of misfolded AAT polymers within hepatocytes [5]. The clinical spectrum of AATD is dominated by pulmonary and hepatic disease, reflecting the dual pathogenic mechanisms. However, the AATD could also determine systemic inflammatory consequences [7] (Table 1).

**Figure 1:**
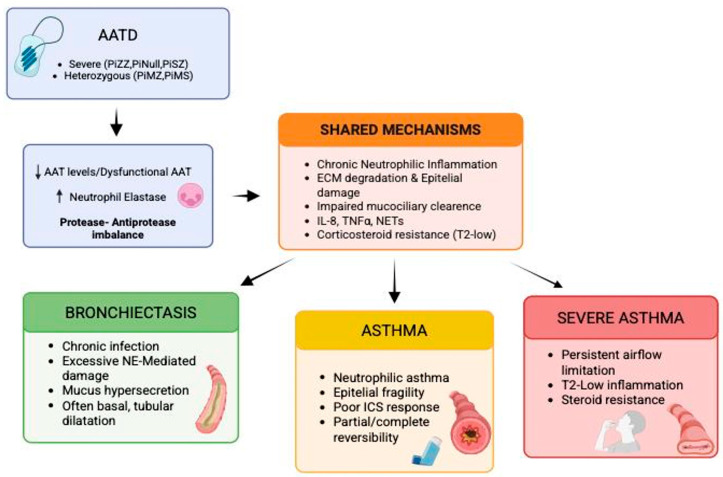
Shared and distinct pathophysiological pathways linking alpha-1 antitrypsin deficiency (AATD) to bronchiectasis, asthma and severe asthma. Abbreviations: AATD, alpha-1 antitrypsin deficiency; AAT, alpha-1 antitrypsin; NE, Neutrophil Elastase; ECM, extracellular matrix; NETs, neutrophil extracellular traps; ICS, inhaled corticosteroids.

While emphysema and COPD represent the classical pulmonary phenotypes of AATD and have been extensively reviewed elsewhere, increasing attention has been directed toward its potential association with other, less common respiratory manifestations. The aim of this review is therefore to provide an updated overview of the evidence linking AATD with respiratory diseases other than COPD and emphysema, particularly focusing on bronchiectasis, asthma, and severe asthma (Figure 1).

### 1.2. Methodology

We performed a literature search in PubMed/MEDLINE for studies published up to 30 September 2025. Search terms included “*alpha-1 antitrypsin deficiency*”, “*AATD*”, “*bronchiectasis*”, “*asthma*”, and “*severe asthma*”. Titles, abstracts, and full texts were screened, including original studies, reviews, and consensus papers relevant to AATD-related airway disease. Non-pertinent articles were excluded.

## 2. Bronchiectasis and AATD

### 2.1. Bronchiectasis

Bronchiectasis is a chronic lung disease defined by irreversible bronchial dilation, typically accompanied by chronic cough and recurrent respiratory infections, and diagnosed by high-resolution computed tomography (HRCT) [8].

Bronchiectasis is associated with a variety of causes, including congenital disorders, acquired insults, and immune/inflammatory conditions, though 10–50% remain idiopathic [9]. The core pathophysiological mechanism involves a self-perpetuating vicious cycle, initiated by epithelial injury leading to impaired mucociliary clearance. 

This promotes chronic bacterial colonization, which in turn triggers neutrophil-dominated inflammation. The subsequent release of proteolytic enzymes causes tissue destruction and progressive bronchial dilation [10]. NE, other serine proteases and matrix metalloproteinases (MMPs) degrade extracellular matrix, disrupt ciliary function, enhance mucus production, and correlate with disease severity and outcomes [11].

A protease–antiprotease imbalance, particularly due to severe AATD (PiSZ, PiZZ), contributes to bronchiectasis pathogenesis [12].

Radiological subtypes (cylindrical, varicose, cystic) reflect severity and chronicity [13], while inflammatory phenotypes vary: neutrophilic is most common, but up to one-third show eosinophilic profiles, relevant for treatment [14].

### 2.2. Epidemiological Evidence: Prevalence of Bronchiectasis in AATD

Although pulmonary emphysema remains the hallmark pulmonary manifestation of AATD, increasing evidence supports a non-negligible prevalence of bronchiectasis in this population. In the recent European Alpha-1 Research Collaboration (EARCO) International Registry, among 418 PiZZ individuals with available imaging, isolated bronchiectasis was present in 9.1% of cases, while 27% exhibited coexistent bronchiectasis and emphysema on HRCT scans. The patients with isolated bronchiectasis were primarily female, never-smokers, and with pulmonary function in the normal range [15].

Earlier work by Parr et al. reported radiological evidence of bronchiectasis in the majority (94%) of 74 subjects PiZ, of which 27% were clinically significant. In most cases bronchiectasis was either tubular or tubulo-varicose, less frequently with cystic morphology. Also subjects with greater bronchiectasis severity had more severe emphysema [16]. The prevalence of bronchiectasis in AATD appears to correlate with genotype severity, supporting the link between AATD and structural airway damage. In a cohort study comparing 1290 patients with severe and intermediate genotypes (PiZZ, PiSZ, PiMZ), bronchiectasis was identified in 20.9% of PiZZ individuals with no link to lung function. These data suggest that the risk of bronchiectasis is not uniform across genotypes, but rather amplified in those with more profound AATD, consistent with the greater degree of protease–antiprotease imbalance [17]. In a recent review, Sanduzzi et al. highlighted that the prevalence of bronchiectasis among individuals with AATD is highly variable, suggesting a true association between the two conditions rather than a mere coincidence [18].

Conversely, when assessing the prevalence of AATD among patients with bronchiectasis, data suggest that the condition is relatively rare but not negligible. In a large multicenter UK cohort including over 1600 patients with bronchiectasis, Carreto et al. reported that routine screening identified severe alpha-1 antitrypsin deficiency in only 0.5% of cases [19]. Also, analysis from the European Multicenter Bronchiectasis Audit & Research Collaboration (EMBARC) registry reported that while AATD is an uncommon cause of bronchiectasis among unselected patients, but under-testing may underestimate its prevalence [20].

In summary, the reported prevalence of bronchiectasis in AATD is variable and increases in association with emphysema. Risk is highest among patients with severe genotypes, particularly PiZZ. Conversely, while AATD is relatively rare among bronchiectasis cohorts, its clinical relevance justifies targeted screening, particularly in idiopathic or early-onset forms.

### 2.3. Pathophysiology: How Excess NE Damages the Bronchial Wall Beyond the Parenchyma

Neutrophilic inflammation plays a pivotal role in the pathogenesis of bronchiectasis associated with AATD. In AATD, the lack of functional AAT permits unopposed NE activity, which not only damages alveolar structures but also affects the bronchial wall. NE degrades key extracellular matrix proteins—including elastin, collagen, and fibronectin—weakening airway integrity and promoting bronchial wall injury (Table 2) [21].

Beyond structural degradation, NE impairs mucociliary clearance, increases epithelial permeability, and stimulates mucus hypersecretion, as well as pro-inflammatory cytokines such as IL8 and TNFα, promoting a cycle of infection and inflammation—hallmarks of bronchiectasis [10,22]. In addition to antiprotease activity, AAT also exerts anti-inflammatory effects by modulating cytokine production and inflammasome activation; thus, its deficiency facilitates a chronic neutrophilic inflammatory phenotype within the airways [5]. Evidence from bronchoalveolar lavage and sputum studies demonstrates elevated NE, IL-8, and proteinase 3 levels in AATD patients, even among those without emphysema [23]. Moreover, NETs formation, a process associated with tissue injury and microbial persistence, appears exaggerated in AATD, contributing further to airway remodeling [24].

**Figure 2:**
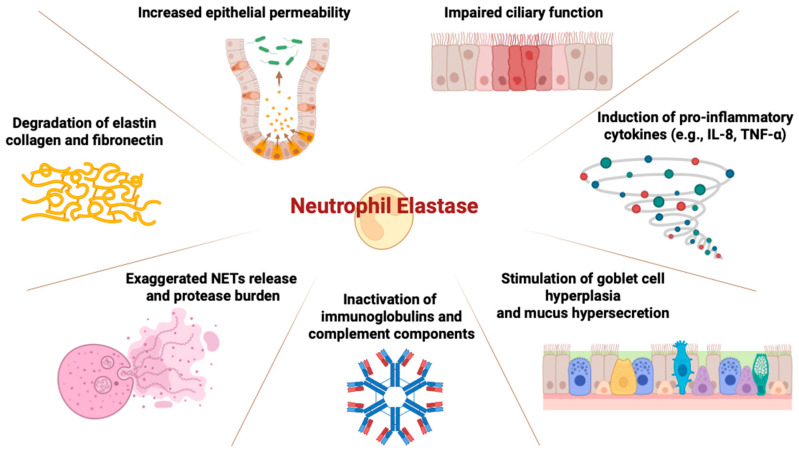
The central role of Neutrophil Elastase (NE) in the pathophysiology of AATD-related bronchiectasis. Abbreviations: AATD, alpha-1 antitrypsin deficiency; NE, neutrophil elastase;IL-8, Interleukin-8; TNF-alpha, tumor necrosis factor-alpha; NETs, neutrophil extracellular traps.

These findings support the view that, in AATD, bronchiectasis can emerge not merely as a consequence of infection or emphysema but as a primary manifestation of dysregulated neutrophilic inflammation (Figure 2) [22].

### 2.4. Bronchiectasis Phenotypes Associated with AATD

Although bronchiectasis in AATD has not been universally defined by a pathognomonic radiological pattern, several studies suggest that certain imaging features may be more prevalent or characteristic in this specific population. Some HRCT studies have reported a predominant basal and lower lobe distribution of bronchiectasis in AATD, in contrast to the more diffuse or upper-lobe patterns typically observed in idiopathic or post-infectious bronchiectasis [15,16,25]. 

**Figure 3:**
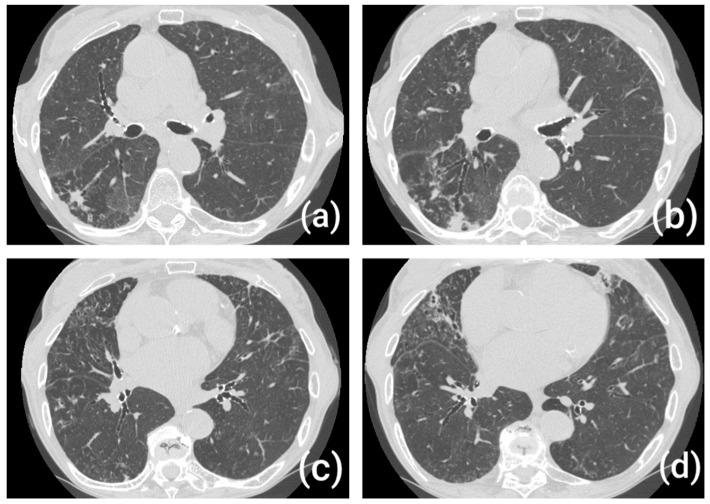
(**a**–**d**) HRCT scans with tubular and predominant basal bronchiectasis in AATD patient. Abbreviations: HRCT, high-resolution computed tomography.

The most common morphological subtype seen in AATD is cylindrical (tubular) bronchiectasis, as opposed to cystic or varicose forms. This may reflect a more subtle and progressive inflammatory process, in contrast to acute destructive damage seen in post-infectious etiologies [15,16,17,26] (Figure 3).

In individuals with severe AATD (PiZZ), bronchiectasis frequently coexists with panacinar emphysema [16]. Data from the EARCO registry show that among PiZZ patients with emphysema, over a quarter also exhibit bronchiectatic changes, while isolated bronchiectasis is less common. This association suggests a common pathophysiological mechanism of proteolytic tissue injury affecting both alveoli and airways. Recognizing this overlap is clinically relevant, as it may influence disease severity, microbial colonization, and therapeutic strategies [15].

### 2.5. Serum AAT Levels, Bronchiectasis Severity and Therapeutic Implications

Observational data suggest that significantly deficient levels of AAT are associated with both emphysematous and airway predominant phenotypes [26].

Although augmentation therapy (AT) is primarily indicated for emphysema, some evidence supports its potential anti-inflammatory effects in the airways, including reductions in NE activity and IL-8 levels [27,28]. Even if no randomized trials have specifically assessed AT efficacy in AATD-related bronchiectasis, observational data suggest possible clinical benefit in selected patients with bronchiectasis [27].

Nevertheless, the European Respiratory Society (ERS) does not recommend AT in bronchiectasis citing insufficient evidence of clinical efficacy and lack of data demonstrating a reduction in exacerbations [29].

### 2.6. Clinical Implications

Despite the well-established role of AATD in pulmonary disease, current guidelines from the European Respiratory Society (ERS, 2017) and the British Thoracic Society (BTS, 2019) do not recommend routine screening for AATD in all patients with bronchiectasis, citing the low prevalence of severe genotypes in unselected cohorts and limited evidence of clinical impact in this subgroup [30,31].

In contrast guidelines for AATD supported by the Alpha-1 Foundation suggest screening all patients with unexplained bronchiectasis for AATD [32].

Moreover, early identification of AATD in bronchiectasis has important clinical implications, including genetic counseling, smoking cessation support, and the consideration of AT in specific clinical cases [33].

Thus, while universal screening in bronchiectasis is not currently recommended, a selective approach based on clinical, radiological, or familial features may improve diagnostic accuracy, allow earlier and more individualized disease monitoring, and refine prognostic stratification in this heterogeneous population. Importantly, the identification of AATD may also influence clinical management even in the absence of AT, prompting a more aggressive approach to risk factor modification, closer functional and microbiological follow-up, and refined prognostic stratification in this heterogenous disease. Moreover, early recognition of AATD gains additional relevance in light of the development of novel therapeutic approaches for bronchiectasis that are currently emerging [34].

## 3. Asthma and AATD

### 3.1. Asthma

Asthma is a heterogeneous chronic airway disease characterized by variable respiratory symptoms (wheeze, dyspnea, chest tightness, cough) and variable airflow limitation on objective testing (spirometry/PEF: post-bronchodilator FEV_1_ increase ≥12% and ≥200 mL; PEF variability >12%) [35].

Clinical heterogeneity spans from allergic to non-eosinophilic, cough-variant, adult-onset, and obesity-related phenotypes, and a subset of patients develops not-fully reversible airflow limitation over time [35]. Etiology is multifactorial: childhood-onset asthma is strongly linked to the 17q21 locus (ORMDL3/GSDMB), whereas adult-onset asthma shows HLA associations and a greater influence of environmental factors (pollutants, tobacco/vaping, wildfire smoke, psychosocial stressors), with recognized exposure–virus interactions; early-life microbiome features and epigenetic modifications further modulate risk and treatment response, limiting the predictive power of polygenic scores [36,37]. Biologically, a type-2 immune response (IL-4, IL-5, IL-13) with mast-cell and eosinophil activation occurs alongside epithelial fragility; chronic inflammation sustains airway remodeling (basement membrane thickening, airway smooth muscle hyperplasia, goblet-cell metaplasia) and bronchial hyperresponsiveness [38]. In non-eosinophilic and steroid-refractory phenotypes, a protease–antiprotease imbalance is increasingly implicated: reduced AAT quantity or function permits excessive neutrophil elastase activity, promoting epithelial injury, extracellular-matrix degradation, mucus hypersecretion, and neutrophil-driven inflammation, potentially contributing to fixed airflow limitation and aligning mechanistically with Alpha-1 antitrypsin deficiency across airway diseases [39]. This biological complexity suggests that additional systemic or genetic modifiers—such as AAT deficiency—may influence specific asthma phenotypes and treatment responses.

### 3.2. Epidemiological Evidence: Prevalence of Asthma in AATD

The association between AATD and asthma has been explored across different populations, yielding heterogeneous results largely due to variations in diagnostic criteria, genotypes analyzed, and patient selection. Reported frequencies of AATD variants (e.g., PiZZ, PiS) among asthmatic patients range from approximately 3% to 25%, while the prevalence of asthma among individuals with AATD fluctuates between 1.4% and 44.6%, depending on national registries and definitions adopted [40]. These wide discrepancies likely reflect selection biases and the lack of standardized testing protocols. Severe deficiency (homozygous PiZZ) remains rare in the general asthmatic population, whereas heterozygous forms (PiMZ and PiSZ) are encountered more frequently—particularly among patients with persistent airflow limitation, late-onset disease, or non–type 2 asthma phenotypes [40]. Although a direct causal relationship has not been established, the coexistence of AATD and asthma appears enriched in specific clinical subgroups characterized by neutrophilic airway inflammation, poor corticosteroid response, or coexisting bronchiectasis—features that often fall within the asthma–COPD overlap (ACO) spectrum [41].

Most available data derive from cross-sectional studies with small sample sizes, unconfirmed genotyping, and variable definitions of asthma severity, which limit the comparability of results. Nevertheless, additional evidence supports the relevance of targeted testing. In a recent analysis by Rahaghi and colleagues, AAT deficiency was identified in approximately 0.6% of patients with asthma and incompletely reversible airflow obstruction—an apparently small yet clinically meaningful prevalence, given the simplicity of testing and the potential therapeutic implications. These findings reinforce the value of selective AATD screening in adults with persistent obstruction, unexplained lung function decline, or atypical asthma phenotypes [42]. 

In summary, the association between AATD and asthma does not involve all patients but represents a clinically relevant entity within well-defined subgroups, where timely identification may refine both diagnosis and management, paving the way for personalized treatment and appropriate genetic counseling. At present, however, no formal recommendation for systematic screening exists in current asthma guidelines, which may contribute to underdiagnosis in this setting [40,41].

### 3.3. Common Pathophysiological Mechanisms

**Figure 4:**
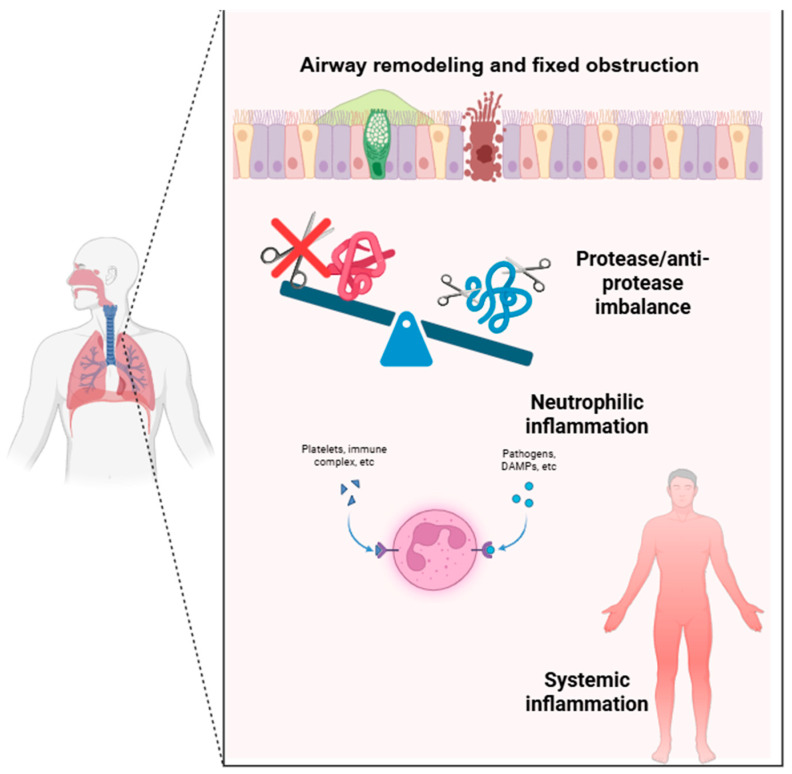
Pathophysiological mechanisms linking AATD and asthma.

Although asthma and AATD are distinct clinical entities, they share several pathophysiological mechanisms, particularly in non-T2 or steroid-resistant asthma phenotypes, as summarized in Figure 4:

a. In individuals with AATD, the deficiency of AAT—the main inhibitor of NE—leads to unregulated proteolytic activity, resulting in degradation of the extracellular matrix and bronchial epithelium; the damaged bronchial epithelium becomes more permeable to allergens, pathogens, and irritants, enhancing airway hyperresponsiveness and sustaining inflammation. Similarly, elevated levels of NE have been observed in severe neutrophilic asthma, suggesting that an imbalance between proteases and antiproteases may represent a shared mechanism [41,43].
b. Beyond its antiprotease activity, AAT also exerts anti-inflammatory and antioxidant effects: it modulates the production of TNF-α, IL-6, and IL-1β by macrophages; limits epithelial apoptosis; and protects against autoimmune responses [44]. Its reduction, even partial and not necessarily in the presence of clinically overt AATD, may thus contribute to enhanced pulmonary inflammation. Moreover, Z-Alpha-1 antitrypsin (Z-AAT), in its polymerized form, can acquire pro-inflammatory and neutrophil chemoattractant properties. Bronchial epithelial cells and type II alveolar cells express AAT; in patients with Z-AATD, these cells may accumulate and secrete Z-AAT polymers, leading to their deposition in the submucosa of the airways. The pleiotropic actions of AAT on inflammation and immunity—including inhibition of IgE-mediated histamine release from mast cells and reduction in eosinophil counts—may also contribute to its association with asthma [41].
c. Persistent inflammation and protease activity induces goblet-cell metaplasia, airway smooth muscle hyperplasia, and subepithelial fibrosis. These changes thicken the airway wall and impair mucociliary clearance, promoting mucus retention and airflow obstruction. Over time, these structural alterations may culminate in a phenotype of fixed obstruction or asthma–COPD overlap (ACO), even in patients without radiological emphysema.
  This mechanism may also explain why heterozygous AATD carriers (PiMZ, PiSZ) often exhibit progressive lung function decline similar to that observed in non–type 2 asthmatic patients [45,46].
d. Neutrophilic airway inflammation is characteristically less responsive to corticosteroids than eosinophilic inflammation. This reduced sensitivity is linked to decreased glucocorticoid receptor expression, oxidative stress, and protease-mediated degradation of anti-inflammatory mediators. Consequently, AAT deficiency may not only promote airway inflammation but also reduce responsiveness to conventional corticosteroid therapy, contributing to persistent airflow limitation and disease progression [46,47].
e. The chronic systemic inflammation associated with AAT deficiency—marked by elevated IL-6 and IL-17 signaling—can extend beyond the lungs, contributing to comorbid conditions such as chronic rhinosinusitis or bronchiectasis. These diseases, in turn, can amplify asthma severity, worsen airflow obstruction, and reduce treatment efficacy, creating a bidirectional relationship between local and systemic inflammation [48,49].

### 3.4. Asthma Phenotypes Associated with AATD

The heterogeneity of asthma manifests in a variety of clinical and inflammatory phenotypes, some of which may overlap with the consequences of AATD. T2-low subtypes, characterized by late-onset asthma, persistent airflow obstruction, and suboptimal response to corticosteroids, appear to be the most closely associated with AATD. For instance, some studies suggest that heterozygous carriers (e.g., PiMZ and PiSZ genotypes) exhibit a decline in lung function and airflow limitation similar to that seen in non-T2 asthmatic patients with persistent obstruction [41,50].

The literature also indicates that patients with AATD (including heterozygotes) frequently present with neutrophilic pulmonary inflammation—a typical pattern in T2-low phenotypes—which includes incompletely reversible airflow obstruction [40,41].

### 3.5. Clinical Implications

Although the current Global Initiative for Asthma (GINA) guidelines for asthma management do not include systematic screening for AATD, the joint ATS/ERS 2003 recommendations on AATD suggest that AAT levels should be measured in all adult patients with incompletely reversible airflow obstruction, regardless of the final clinical diagnosis (COPD, asthma, or other) [51]. This divergence between guidelines may contribute to the underdiagnosis of AATD in asthmatic patients, particularly those with atypical features, T2-low phenotypes, or a clinical course refractory to standard therapy [52]. Identifying AATD not only helps clarify the causes of persistent airflow obstruction or poor responsiveness to corticosteroids but also allows for the implementation of targeted management strategies, such as early smoking cessation, avoidance of harmful environmental exposures, and regular monitoring of lung function [32,53]. In patients with severe deficiency (e.g., PiZZ genotype), the diagnosis has important prognostic implications and, in selected cases, may open the door to disease-specific treatments such as augmentation therapy with AAT, which is currently approved for emphysema but under investigation for use in asthma with fixed airflow limitation [32]. 

A multicenter study conducted in Colombia identified clinically relevant SERPINA1 gene mutations in 12.5% of patients with difficult-to-treat asthma, suggesting a non-negligible prevalence of AATD in this population and reinforcing the importance of targeted screening in selected cases [54].

Overall, incorporating AAT testing into the evaluation of asthmatic patients with non-T2 phenotypes, fixed obstruction, or atypical clinical presentations may represent a step toward more personalized and timely management of underlying comorbid conditions.

## 4. Severe Asthma and AATD

### 4.1. Severe Asthma

Severe asthma affects approximately 3.5–10% of the asthmatic population. According to both GINA and ERS/ATS it is defined as asthma that remains uncontrolled despite good adherence to optimized high-dose inhaled corticosteroids (ICS) plus a second controller, and management of contributory factors, or that worsens when such treatment is reduced [55,56]. However, there are some differences between GINA and ERS/ATS as summarized in Table 3.

The pathophysiology of severe asthma is quite complex and implies interactions between immune, structural, and environmental mechanisms that induce persistent airway inflammation, remodeling, and consequently variable responses to therapy [57,58]. Two distinct inflammatory endotypes are recognized:

*a.*  T2-high (eosinophilic asthma), driven by IL-4, IL-5, IL-13, and IL-33, is associated with airway eosinophilia, elevated FeNO, and responsiveness to biologics targeting IL-5 or IL-4/IL-13 pathways [59,60].
*b.*  T2-low asthma, often neutrophilic or paucigranulocytic, involves IL-17, IL-8, and CXCR2 pathways and is frequently resistant to corticosteroids [59,61].

Inflammation may persist despite high-dose ICS or systemic corticosteroids through mechanisms such as reduced histone deacetylase (HDAC2) activity, altered glucocorticoid receptor function, oxidative stress and activation of pro-inflammatory transcription factors such as NF-κB and AP-1 [57,58]. Airway epithelial cells release alarmins (TSLP, IL-25, IL-33) that activate dendritic cells, ILC2s, and eosinophils [55,58]. Persistent inflammation leads to structural changes such as: subepithelial fibrosis, smooth muscle hypertrophy, goblet cell hyperplasia, and angiogenesis. These changes bring to fixed airflow limitation and reduced bronchodilator responsiveness [58,62]. Remodeling is consistent in patients with long-standing disease and contributes to the “irreversible” component of airflow obstruction [57,60]. Patients commonly experience frequent exacerbations triggered by viral infections, allergens, or pollutants [61,62]. Given the heterogeneous inflammatory pathways involved, genetic and protease–antiprotease imbalances, such as those observed in alpha-1 antitrypsin deficiency (AATD), have been proposed as potential modifiers of severe asthma pathobiology [41,63].

### 4.2. Epidemiological Evidence and Pathophysiological Mechanisms of Asthma in AATD

Across cohorts of patients with severe or difficult-to-treat asthma, clinically significant AATD (PiZZ or PiNull) is consistently uncommon, generally accounting for less than 1% of total cases. Conversely heterozygous variants (PiMZ, PiSZ) are more frequent with reported prevalences ranging from 5 to 20%, depending on cohort selection, disease severity, and testing strategy [63,64,65,66,67]. The prevalence estimates that are reported varied widely across studies, reflecting differences in cohort selection and testing strategies, including serum AAT measurement alone versus confirmatory phenotyping or genotyping.

Alpha-1 antitrypsin deficiency (AATD) has been investigated as a potential modifier in severe or difficult-to-treat asthma (especially T2-low asthma), as both conditions share biological features that can lead to persistent airflow limitation and poor disease control. These include the following:

a. Protease–antiprotease imbalance, with reduced levels of AAT leading to increased NE activity [63,64,66].
b. Neutrophilic airway inflammation, that is associated with corticosteroid resistance and worse outcomes [7,65].
c. Incomplete reversibility of airflow obstruction, common to severe asthma and AATD-related emphysema [64,66,67].

This overlaps justify investigation of AATD in asthma populations with atypical or severe phenotypes [67]. Table 4 summarizes major studies evaluating AATD in severe asthma.

Early studies by van Veen (2006) and Mousavi (2013) showed no significant relationship between heterozygous AATD and fixed obstruction [64,65]. The prevalence of non-PiM genotypes (≈5%) was similar to the general population, and no association was found with lung function decline. However, a more recent work by Vianello et al. conducted in 2021 provides new insight as they have identified AATD in ≈7% of biologic-treated severe asthmatics, all heterozygotes, who exhibited a greater annual decline in FEV_1_ and FVC despite optimal therapy in comparison to control group (patients without AATD) [66]. In addition non-smoker patients with AATD presented a significantly more severe lung decline. In 2024 Zappa et al. confirmed these observations: 19% of GINA Step 5 patients enrolled had non-MM genotypes, associated with lower AAT levels, higher emphysema prevalence, and poorer improvement in asthma control and in eosinophilic inflammation during follow up in comparison with PiMM individuals. Moreover non-MM patients at the baseline presented a higher number of neutrophils in induced sputum [67]. Navasero et al. (2024) further demonstrated the feasibility and clinical yield of systematic AATD screening among moderate-to-severe asthmatics eligible for biologics, detecting ≈ 10% heterozygous carriers (compared to 0.02% among general population) [68].

Additional evidence on AATD in asthma cohorts with mixed severities, including patients with severe asthma, is listed in Table 5. These have increasingly suggested a potential link between AATD and specific asthma phenotypes, particularly those characterized by poor control and frequent exacerbations.

Eden et al. (2007) found ≈10% carriers and 2–3% with low AAT levels among poorly controlled asthmatics [69]. This observation raised the hypothesis that genetic variation in *SERPINA1* might contribute to the heterogeneity and severity of asthma beyond traditional inflammatory pathways. Building on this, Suárez-Lorenzo et al. (2018) explored the distribution of alpha-1 antitrypsin levels in allergic asthmatic individuals sensitized to house dust mites. Their findings revealed altered AAT levels even in this well-defined allergic population [70]. More recently, Martín-González et al. (2025) provided robust genetic evidence that the PiS* and PiZ* variants of the *SERPINA1* gene are significantly associated with an increased risk of asthma exacerbations [71]. Consistent with these findings, Ortega et al. (2025) demonstrated that *SERPINA1* genetic variation correlates not only with asthma exacerbation frequency but also with related healthcare utilization. Their results reinforce the idea that AATD-related genotypes may identify a subset of patients with higher morbidity, who might benefit from more personalized management strategies [72,73].

Taken together, these studies consistently point to a meaningful intersection between AATD and asthma pathobiology. They highlight the need for increased awareness and possibly routine screening for *SERPINA1* variants in patients with difficult-to-treat or exacerbation-prone asthma, as genetic background may contribute to disease persistence and therapeutic response.

Across available studies, AATD is uncommon among asthmatics, with heterozygous forms detected in ≈5–10% of severe or biologic-treated cases. Early investigations suggested no major functional effect, but recent data indicate that AATD may accelerate lung function decline or limit therapeutic benefit in selected subgroups. The overall evidence supports viewing AATD as a modifier of disease expression and progression, particularly in neutrophilic or treatment-resistant phenotypes, rather than a direct cause of asthma. From a clinical perspective, targeted screening for AATD should be considered in patients with severe, atypical, or poorly controlled asthma—especially when fixed obstruction, emphysema, or unexplained lung-function decline persists despite optimal therapy.

### 4.3. Clinical Implications

The identification of alpha-1 antitrypsin deficiency (AATD) in patients with severe or poorly controlled asthma may hold relevant clinical implications. Screening for AATD can be particularly useful, as the presence of allelic mutations may influence both disease monitoring and long-term follow-up [67,68]. Therefore, it is advisable to consider testing for AATD—through measurement of serum AAT levels and genotyping—in patients with severe or atypical asthma, especially when fixed airway obstruction or poor therapeutic response is observed. Furthermore, genetic variants such as PiS and PiZ could potentially serve as biomarkers for exacerbation risk stratification, helping to identify subgroups of asthmatics who may experience more frequent or severe exacerbations [66,71,72,73]. Although augmentation therapy has not demonstrated proven efficacy in severe asthma, recognizing AATD in these patients may still impact clinical management. Early identification can refine prognosis, prompt closer surveillance of airflow decline, and encourage more personalized therapeutic approaches [66,67].

In 2008, a case report described a young Caucasian woman with severe asthma and heterozygous AAT deficiency (MZ phenotype) who, despite optimized therapy, experienced rapid functional decline and corticosteroid-related adverse effects. Compassionate intravenous AAT augmentation therapy stabilized lung function, reduced hospitalizations, and allowed steroid withdrawal, markedly improving her quality of life [74]. Although such therapy is not approved for asthma or MZ phenotypes, this observation supports the hypothesis that protease–antiprotease imbalance may contribute to airway inflammation and disease progression, warranting further research in selected severe asthma populations. Recognizing AATD in these patients may still impact clinical management. Early identification can refine prognosis, prompt closer surveillance of airflow decline, encourage smoking cessation and more personalized therapeutic approaches [66,67,68,71,72,73,75].

## 5. Practical Diagnostic Pathway and Clinical Considerations

To enhance the real-world applicability of AATD screening in airway diseases, a structured diagnostic approach is required. Clinical and radiological triggers for testing should include all patients with idiopathic or unexplained bronchiectasis, particularly those with a basal distribution, and patients with severe or difficult-to-treat asthma, especially if characterized by fixed airflow obstruction or an unexpectedly rapid decline in lung function. The diagnostic process should ideally begin with the measurement of serum AAT levels combined with C-reactive protein (CRP). Since AAT is an acute-phase reactant, its levels may rise during infections or exacerbations, potentially masking a deficiency (false-negative results). If CRP is elevated, serum AAT levels should be interpreted with caution and re-evaluated during clinical stability. If serum AAT levels are below the laboratory threshold (typically <110 mg/dL or 1.1 g/L), reflex phenotyping (isoelectric focusing) or genotyping is mandatory to identify the specific allelic variants. In cases of persistent discrepancy between very low AAT levels and common genotypes, gene sequencing (Next-Generation Sequencing, NGS) should be considered to detect rare or null variants. This stepwise approach ensures accurate identification and avoids underdiagnosis in the complex landscape of non-COPD airway diseases.

## 6. Conclusions

AATD should be recognized as a complex condition whose clinical impact extends beyond emphysema and COPD to include even bronchiectasis, asthma and serve asthma. Although emerging evidence supports this broader spectrum, current understanding is constrained by limited, heterogeneous studies and the absence of standardized diagnostic pathways. Improved screening strategies and molecular biomarkers may enhance early recognition and patient stratification. Advancing an integrated clinical, functional, and molecular perspective will be essential to refine the characterization of AATD-related airway disease and to optimize targeted management across chronic respiratory conditions.

## 7. Current Limitations and Future Directions

Despite increasing recognition of the interaction between AATD and chronic airway diseases, the current evidence remains limited. Most studies exploring the link between AATD and bronchiectasis or asthma rely on small observational cohorts, registry analyses, or retrospective imaging reviews. The molecular and cellular mechanisms underlying bronchial destruction—as opposed to alveolar injury—such as protease–antiprotease imbalance, neutrophilic inflammation, and immune dysregulation, remain incompletely understood. Similarly, especially in heterozygous or mildly deficient individuals, whether AATD predisposes to specific inflammatory endotypes, such as T2-low asthma, or modifies treatment response through persistent neutrophilic inflammation, remains an open question. The potential impact of AATD on airway hyperresponsiveness, mucus dynamics, and remodeling processes also requires deeper investigation. From a diagnostic perspective, the absence of standardized criteria or predictive clinical–radiological features to guide AATD testing contributes to significant underdiagnosis across airway diseases. The development of validated screening algorithms, supported by biomarker-driven and genetic approaches, could improve early detection and disease stratification. Therapeutically, the role of augmentation therapy in bronchiectasis and asthma remains uncertain, with no randomized controlled trials addressing its effects on exacerbation burden, bacterial colonization, or radiologic progression. Moreover, potential interactions between augmentation therapy and biologic agents used in severe asthma represent an unexplored but clinically relevant field. Future research should prioritize large-scale, multicenter, endotype-stratified studies integrating clinical, functional, imaging, and molecular data. Such an approach will be crucial to define the true burden of airway disease associated with AATD, elucidate the mechanisms linking protease imbalance to distinct airway phenotypes, and ultimately guide the development of precision diagnostic and therapeutic strategies that extend beyond emphysema and COPD.

### Augmentation Therapy in Non-Emphysematous Airway Disease: Current Evidence and Challenges

Although alpha-1 antitrypsin (AAT) augmentation therapy is an established treatment for emphysema associated with AATD, its role in non-emphysematous airway diseases remains poorly defined. To date, no adequately placebo-controlled randomized clinical trials have specifically evaluated the efficacy of augmentation therapy in patients with AATD-related bronchiectasis, asthma, or severe asthma without emphysema. Available evidence is limited to case reports and small case series, suggesting that selected patients with AATD-associated bronchiectasis may experience subjective clinical improvement and reduced exacerbation frequency following augmentation therapy. However, these observations lack validation in prospective studies. More recently, an exploratory randomized cross-over study (BATMAN trial, NCT05582798) has investigated intravenous AAT augmentation in patients with bronchiectasis and elevated neutrophil elastase activity. This proof-of-concept trial, characterized by a small sample size and short treatment periods, is primarily focused on biological endpoints, including airway inflammation and neutrophil function, rather than clinically meaningful outcomes such as exacerbation rates, lung function decline, or quality of life. While this biomarker-driven approach represents an important step toward personalized treatment strategies, it does not provide definitive evidence of clinical efficacy. Several factors currently limit the development of well-designed clinical trials in this field, including the rarity of severe AATD without emphysema, the heterogeneity of airway disease phenotypes, challenges in patient recruitment, high treatment costs, and uncertainty regarding optimal patient selection and treatment strategies. Moreover, the lack of validated biomarkers restricts the possibility of identifying those patients that most likely could benefit from augmentation therapy. Future research should prioritize adequately powered, multicenter randomized controlled trials enrolling well-characterized patients with documented protease–antiprotease imbalance and clinically relevant endpoints. Such studies are essential to clarify the therapeutic role of AAT augmentation in non-emphysematous airway disease.

## Figures and Tables

**Table 1:** Multisystem clinical manifestations of AATD and underlying pathophysiology.

	Clinical Manifestations	Pathophysiology
Pulmonary	Early-onset emphysema (panacinar, lower lobes)	Loss of function: protease–antiprotease imbalance and neutrophil elastase–mediated tissue damage
	COPD with chronic bronchitis and airflow limitation	
	Asthma/asthma-like symptoms; severe asthma overlap	
	Bronchiectasis	
	Increased risk of respiratory infections	
Hepatic	Neonatal cholestasis	Toxic gain-of-function: hepatocyte injury from accumulation of misfolded AAT polymers
	Chronic hepatitis	
	Cirrhosis	
	Hepatocellular carcinoma	
Other	Panniculitis (rare, necrotizing, painful)	Systemic inflammatory consequences of AATD
	ANCA-associated vasculitis (e.g., GPA)	
	Possible ↑ cardiovascular risk	
	Occasional renal/autoimmune associations	

Abbreviations: AATD, alpha-1 antitrypsin deficiency; AAT, alpha-1 antitrypsin; COPD, chronic obstructive pulmonary disease; ANCA, anti-neutrophil cytoplasmic antibodies; GPA, Granulomatosis with polyangiitis.

**Table 2:** Airway domains affected by NE activity in AATD and downstream consequences.

Domain	Effect of NE Activity	Consequences in AATD
Structural damage	Degradation of elastin, collagen, fibronectin	Airway wall weakening, bronchial dilatation, emphysema
Mucosal barrier	Increased epithelial permeability	Facilitated pathogen invasion and inflammation
Mucociliary clearance	Impaired ciliary function; degradation of opsonins and surfactant proteins	Reduced bacterial clearance, chronic colonization
Mucus production	Stimulation of goblet cell hyperplasia and mucus hypersecretion	Airway obstruction, infection risk
Inflammatory signaling	Induction of pro-inflammatory cytokines (e.g., IL-8, TNF-α)	Neutrophil recruitment, persistent inflammation
Immune modulation	Inactivation of immunoglobulins and complement components	Impaired host defense
Tissue remodeling	Exaggerated NETs release and protease burden	Ongoing bronchial wall injury and remodeling

Abbreviations: NE, neutrophil elastase; AATD, alpha-1 antitrypsin deficiency; IL-8, Interleukin-8; TNF-alpha, tumor necrosis factor-alpha; NETs, neutrophil extracellular traps.

**Table 3:** Definitions of severe asthma: comparison between GINA (2024) and ERS/ATS (2014).

Aspect	GINA (2024)	ERS/ATS (2014)
Core definition	Severe asthma is asthma that remains uncontrolled despite high-dose ICS–LABA and management of contributory factors, or that requires high-dose ICS–LABA to maintain control.	Severe asthma is asthma that requires high-dose ICS plus a second controller and/or systemic corticosteroids to prevent it from becoming uncontrolled, or that remains uncontrolled despite this therapy.
Criteria for uncontrolled asthma	Uncontrolled if symptoms/exacerbations persist despite optimized treatment and exclusion of modifiable factors (e.g., adherence, inhaler technique, comorbidities).	Uncontrolled if ≥1 of the following: • Poor symptom control (ACQ > 1.5 or ACT < 20) • ≥2 severe exacerbations/year • ≥1 hospitalization or ICU admission • Persistent airflow limitation (FEV_1_ < 80% predicted, FEV_1_/FVC < LLN).
Treatment step	High-dose ICS–LABA as minimum requirement.	High-dose ICS plus second controller (usually LABA) and/or systemic corticosteroids.
Perspective	Definition is retrospective, confirmed after several months of optimized therapy and assessment.	Definition is retrospective, but incorporates explicit, objective criteria for uncontrolled asthma.
Focus	Emphasis on identifying and correcting modifiable factors (adherence, inhaler technique, comorbidities).	Emphasis on measurable clinical and functional outcomes (exacerbations, lung function, control scores).

Abbreviations: GINA, Global Initiative for Asthma; ERS, European Respiratory Society; ATS, American Thoracic Society; ICS, inhaled corticosteroids; LABA, long-acting β2-agonist; ACQ, Asthma Control Questionnaire; ACT, Asthma Control Test; ICU, Intensive Care Unit; FEV_1_, forced expiratory volume in 1 s; FVC, forced vital capacity; LLN, lower limit of normal.

**Table 4:** Evidence on AATD in severe asthma cohorts.

Author/Year	Country	Design	Population	AATD Findings	Key Result
van Veen, 2006 [64]	Netherlands	Case–control	Severe asthma with/without fixed obstruction	Non-PiM genotypes	No increased risk of persistent obstruction
Mousavi, 2013 [65]	Iran	Cross-sectional	43 severe persistent asthma	Low AAT in 4.7%	No association with lung function
Vianello, 2021 [66]	Italy	Letter/Clinical report	Severe asthma on biologics	AATD noted in some cases	Possible determinant of poor control and decline
Zappa, 2024 [67]	Italy	Retrospective cohort	GINA step 5 severe asthma	19% non-MM genotypes; lower AAT	More emphysema; clinical response only in PiMM
Navasero, 2024 [68]	USA	Abstract/Screening study	Moderate-severe asthma (on/eligible for biologics)	Feasibility of AATD screening	Showed value of testing in this group

Abbreviations: AATD, alpha-1 antitrypsin deficiency; AAT, alpha-1 antitrypsin; Pi, protease inhibitor allele (PiMM = normal genotype; PiMZ/PiMS = heterozygous carriers; PiZZ/PiSZ = severe deficiency); GINA, Global Initiative for Asthma.

**Table 5:** Evidence on AATD in general asthma cohorts (mixed severities).

Author/Year	Country	Design	Population	AATD Findings	Key Result
Eden, 2007 [69]	USA	Clinical trial cohort	Poorly controlled asthma (ALA-ACRC)	10.5% carriers; 2.4% <20 μM	Detected non-negligible prevalence
Suárez-Lorenzo, 2018 [70]	Spain	Cross-sectional	648 mite-allergic asthmatics (48 severe)	Genotypes & serum AAT	No correlation with severity
Martín-González, 2025 [71]	Spain	Genetic/biomarker	Asthma (severity not specified)	Pi*S, Pi*Z variants; serum AAT	Associated with risk of exacerbations
Ortega, 2025 [72,73]	USA	Genetic association study	Moderate-severe asthma	SERPINA1 variants & serum AAT	Associated with severity via gene-environment interaction

Abbreviations: Pi, protease inhibitor allele (Pi*S/Pi*Z = severe deficiency).

## Data Availability

No new data were created or analyzed in this study.
